# Exploratory microbiota-metabolite alterations associated with cognitive impairment after repeated low-dose sevoflurane exposure in mice

**DOI:** 10.3389/fcimb.2026.1869293

**Published:** 2026-09-30

**Authors:** Hui Tang, Qingqing Xiong, Gang Qian

**Affiliations:** 1 Department of Anesthesiology, Tongren Hospital, Shanghai Jiao Tong University School of Medicine, Shanghai, China; 2 Department of Anesthesiology, Lijiang People’s Hospital, Lijiang, Yunnan, China

**Keywords:** cognitive impairment, gut microbiota, metabolomics, metagenomics, sevoflurane

## Abstract

**Background:**

Repeated low-dose sevoflurane exposure may affect cognition, but the relationship between such exposure, gut microbial composition, and circulating metabolites remains unclear. This study examined an experimental proof-of-concept exposure model and treated microbiota-metabolite findings as exploratory associations.

**Methods:**

Mice were exposed to 100 ppm sevoflurane for 2 h/day over 7 consecutive days, followed by Morris water maze testing without a long-term recovery follow-up. Gut microbiota and serum metabolites were profiled by shotgun metagenomic sequencing and LC-MS metabolomics, respectively, and machine-learning analyses were used only for exploratory feature ranking.

**Results:**

Sevoflurane-exposed mice exhibited poorer Morris water maze performance (Day 6 escape latency: 28.6 ± 3.4 s vs. 12.5 ± 1.8 s in controls, p < 0.01), although interpretation is limited by unequal behavioral sample sizes. Serum metabolomics suggested nominal and exploratory alterations in bile acid- and sphingolipid-related pathways. Shotgun metagenomics (n = 3/group) identified preliminary relative-abundance shifts, including a lower *Bacteroidota/Bacillota* ratio and lower relative abundance of *Akkermansia*.

**Conclusions:**

In this proof-of-concept model, repeated low-dose sevoflurane exposure was associated with impaired spatial learning and memory and with exploratory serum metabolic and gut microbial alterations. The findings do not establish persistent long-term deficits, routine occupational risk, or causal microbiota-metabolite mediation. Larger cage-aware studies, an ambient-air comparison group, targeted validation, and interventional experiments are required.

## Introduction

Inhalation anesthetics are widely used for the induction and maintenance of general anesthesia because of their rapid onset and recovery profiles. However, during routine clinical practice, unavoidable micro-emissions of anesthetic waste gases occur due to equipment leakage, inadequate scavenging systems, and patient exhalation. Despite advances in anesthesia technology, occupational exposure to anesthetic gases remains a persistent clinical concern for operating room personnel (Emara et al., 2020).

Environmental monitoring demonstrates that sevoflurane concentrations vary substantially with ventilation, scavenging, work practices, sampling location, and averaging time. Jafari et al. measured geometric mean concentrations of approximately 0.005 ppm in personnel breathing zones and 0.004 ppm in ambient operating-room air (Jafari et al., 2018), whereas Hua et al. reported a mean concentration of 1.11 ± 0.65 ppm in 172 air samples from 22 operating rooms (Hua et al., 2021). Higher task-related peaks have been documented under less controlled conditions (Braz et al., 2017; Kobos et al., 2024). Regulatory comparisons also require qualification: the US Occupational Safety and Health Administration has no sevoflurane-specific permissible exposure limit, and the often-cited 2 ppm ceiling was a 1977 generic NIOSH recommendation for halogenated anesthetic agents that predated the clinical use of sevoflurane, not a sevoflurane-specific recommended exposure limit (Kobos et al., 2024). The 2024 NIOSH evaluation summarizes selected full-shift or short-term comparison values ranging from 5 to 50 ppm across agencies and countries, but these values use different averaging frameworks (Kobos et al., 2024). Accordingly, none is directly equivalent to a 100 ppm animal-chamber exposure for 2 h/day. The 100 ppm (0.01%) regimen represents an upper-range, sub-anesthetic proof-of-concept exposure that is substantially above typical monitored operating-room concentrations and does not simulate routine occupational exposure.

As one of the most commonly used volatile anesthetics, sevoflurane has been implicated in perioperative cognitive outcomes, including postoperative neurocognitive disorders (PND) (Miller et al., 2018). PND refers specifically to neurocognitive disorders arising in perioperative or postoperative settings. Literature closer to a non-surgical, repeated-exposure design remains limited. Ozer et al. exposed rats to 0.3% sevoflurane for 4 h/day over 30 days and reported behavioral changes (Ozer et al., 2006); although sub-anesthetic and repeated, that concentration was approximately 30-fold higher than the 0.01% used here. Other non-surgical repeated-exposure studies used 1.5-2.5% sevoflurane in aged rats (Guo et al., 2018) or 3% sevoflurane in neonatal mice (Liang et al., 2023) and reported changes involving BDNF/NF-kappaB/apoptosis or sequential tau phosphorylation, respectively. These age-specific, anesthetic-range models are not directly equivalent to the present adult low-concentration model. Oxidative stress, ferroptosis, neuroinflammation, mitochondrial dysfunction, and neuronal apoptosis have been reported in perioperative or anesthetic-range studies (Xie et al., 2015; Ye et al., 2019; Xu et al., 2023; Zhang et al., 2023; Zheng et al., 2024), but these mechanisms were not assessed in the present experiment. The present study instead examined post-exposure cognitive impairment after repeated low-dose sevoflurane without surgery and did not model PND or occupational disease.

Direct evidence linking sevoflurane to the gut microbiota is emerging but remains model-dependent. A single 2.21% sevoflurane exposure altered the murine intestinal microbiome over 14 days (Han et al., 2021). In aged mice, repeated 3% sevoflurane exposure was associated with cognitive impairment, reduced *Akkermansia* abundance, NLRP3 inflammasome activation, and partial improvement with probiotic treatment (Han et al., 2024). A recent developmental model also associated repeated sevoflurane exposure with microbiota changes and reported attenuation after broad-spectrum antibiotic pretreatment, although antibiotic effects are not microbiota-specific (Zhang et al., 2026). These studies used anesthetic-range developmental or aged-animal paradigms and therefore motivate, but do not establish, microbiota-mediated mechanisms in the present 100 ppm adult model.

Metagenomic and metabolomic approaches have identified candidate microbial and metabolic patterns after sevoflurane exposure in several animal models (Han et al., 2021; Han et al., 2024; Zhang et al., 2026). In the present study, serum metabolites and fecal microbial profiles were analyzed to generate associative hypotheses rather than establish causal links between microbiota-derived metabolites and brain dysfunction.

Occupational studies have primarily characterized workplace concentrations or systemic health endpoints (Braz et al., 2017; Jafari et al., 2018; Emara et al., 2020; Hua et al., 2021; Ji et al., 2021; Oliveira et al., 2021; Kobos et al., 2024). Only limited animal literature has examined chronic sub-anesthetic behavioral exposure (Ozer et al., 2006), whereas representative mechanistic and cognitive studies have predominantly used perioperative, developmental, aged, or anesthetic-range paradigms (Xie et al., 2015; Guo et al., 2018; Miller et al., 2018; Ye et al., 2019; Liang et al., 2023; Xu et al., 2023; Zhang et al., 2023; Han et al., 2024; Zheng et al., 2024; Huang et al., 2025). Against this background, the primary unresolved question addressed here is whether repeated 0.01% sevoflurane exposure without surgery is associated with a post-exposure cognitive phenotype. The serum metabolomic and fecal metagenomic analyses are secondary, hypothesis-generating components used to identify co-occurring candidate signals. The study was not designed to reproduce routine occupational exposure or to establish microbiota-metabolite mediation.

In the present study, we established a repeated low-dose sevoflurane (LD-Sev) exposure model as an upper-range proof-of-concept paradigm while avoiding surgical trauma and invasive perioperative confounders. By omitting surgery, this design helps distinguish exposure-associated observations from surgical injury and other perioperative factors that complicate conventional postoperative neurocognitive models. We combined behavioral testing, serum metabolomics, shotgun metagenomics, and exploratory machine-learning feature ranking to characterize co-occurring behavioral, metabolic, and microbial changes across complementary biological levels. The machine-learning analyses ranked candidate serum metabolic features that distinguished the exposure groups within this dataset; This integrated framework identifies testable candidate pathways and provides a focused basis for targeted validation and interventional studies of cognitive dysfunction after repeated sevoflurane exposure.

## Materials and methods

### Sevoflurane-associated cognitive dysfunction model

Male C57BL/6J mice (8 weeks old, 22 ± 2 g) were obtained from the Shanghai Laboratory Animal Center (SLAC, CAS, Shanghai, China). After 7 days of acclimatization, mice were randomly assigned using a random-number table to the normal control (NS) or SCOD group (n = 10 per group). Mice were housed under specific pathogen-free conditions at 22 ± 1 °C and 50 ± 5% humidity under a 12-h light/dark cycle. Five mice were housed per cage, resulting in two cages per group. Bedding, diet, water, environmental enrichment, and daily health monitoring were standardized across groups. All procedures were approved by the Ethics Committee of Shanghai Tongren Hospital (approval no. Tongren Lunshen A2023-130-01).

To establish a repeated low-dose, sub-anesthetic SCOD model without surgical stress, mice were placed in a sealed acrylic exposure chamber (approximately 2 L). The chamber was connected to a calibrated sevoflurane vaporizer and a dual-inlet gas mixer. The target inspired sevoflurane concentration (FiSev) was approximately 100 ppm (0.01%). This upper-range proof-of-concept concentration exceeded typical occupational averages and was selected to facilitate the detection of biological signals. A sevoflurane-containing gas stream was diluted with carrier gas to achieve the target concentration. FiSev was continuously monitored using an anesthetic-gas monitor (QZKJ-C4H3F7O, Qianzhao Technology, China; detection limit, 1 ppm), and gas delivery was adjusted accordingly. Because session-level readings were not electronically archived, FiSev is reported as an approximate target rather than a measured session average.

Mice in the SCOD group were exposed for 2 h daily over seven consecutive days, beginning on day 0. All exposures were conducted at the same circadian time. Control mice underwent identical handling and chamber procedures under the same carrier-gas conditions but without sevoflurane. The nominal fraction of inspired oxygen (FiO_2_) was maintained near 0.40 in both groups. Chamber temperature was maintained at 36.5–37.5 °C, and freshly replaced soda lime was used to maintain carbon dioxide below 0.3%. Outflow gases were actively scavenged.

Spontaneous respiration and the righting reflex were monitored throughout each exposure. Sevoflurane administration would have been discontinued if respiratory depression or loss of the righting reflex occurred. No mouse met these stopping criteria. After each session, the chamber was flushed with carrier gas for 5 min before the mice were returned to their home cages. Body weight, grooming, and general activity were monitored daily. No severe illness, respiratory depression, or loss of the righting reflex was observed. Longitudinal body-weight records were not retained for formal between-group analysis.

Behavioral testing began after completion of the 7-day exposure schedule, without an additional long-term recovery interval. The observed phenotype was therefore interpreted as short-term post-exposure cognitive impairment rather than a persistent long-term deficit. Five of the 10 control mice were randomly selected for Morris water maze (MWM) testing, whereas all 10 SCOD mice underwent behavioral testing. The remaining five control mice were retained as non-behavioral counterparts to ensure adequate serum and fecal material for downstream analyses. The behavioral assessor was blinded to group allocation wherever feasible.

Serum metabolomics was performed in eight mice per group. The control subset included the five behaviorally tested mice and three additional controls. Metagenomic sequencing was performed using fecal samples from three individual mice per group. These samples were selected from the individually collected 24-h fecal samples based on sufficient fecal material and sample quality. Downstream omics analysts were blinded to group allocation wherever feasible.

No invasive sampling was performed before MWM testing, and no additional anesthetic was administered during exposure or behavioral assessment. Fecal samples for metagenomic sequencing were collected individually during the 24 h preceding euthanasia. Terminal serum samples were collected after euthanasia.

Mice were euthanized by gradual-fill carbon dioxide (CO_2_) inhalation in accordance with institutional guidelines for laboratory animal care. The CO_2_ flow rate was maintained at 30–70% of the chamber or cage volume per minute to minimize distress. After loss of consciousness and cessation of respiration and heartbeat were confirmed, cervical dislocation was performed as a secondary method to confirm death.

### Behavioral assessment

Spatial learning and memory were assessed using the Morris water maze (MWM) over seven consecutive days in a circular pool divided into four quadrants (NE, NW, SE, and SW), with the hidden platform fixed in the SW quadrant throughout acquisition. On Day 1, mice underwent visible-platform training; each mouse completed four trials from four release points with 30-min inter-trial intervals. From Days 2 to 6, the platform was submerged 1–2 cm beneath opaque water. Escape latency and path length were recorded using an automated tracking system. On Day 7, a 60-s probe trial was conducted with the platform removed. For hidden-platform acquisition, the four trials for each mouse were averaged within each day, and mouse-level daily means were used as the statistical unit. Acquisition data were analyzed using two-way repeated-measures ANOVA (group × day) with Greenhouse-Geisser correction followed by multiple-comparison *post hoc* tests where appropriate, and probe-trial outcomes were compared using unpaired two-tailed t-tests (GraphPad Prism v10.1; α = 0.05).

### Metabolomics procedures

#### Sample preparation and LC-MS acquisition

Serum (100 μL) was added to 300 μL of pre-chilled methanol/acetonitrile/water (2:2:1, v/v/v), vortexed, sonicated for 30 min at low temperature, precipitated at -20 °C for 10 min, and centrifuged at 14,000 × g for 20 min at 4 °C. The supernatant was vacuum-dried and reconstituted in 100 μL acetonitrile/water (1:1, v/v). Chromatographic separation was performed using a Waters ACQUITY UPLC HSS T3 column (100 mm × 2.1 mm, 1.7 μm) maintained at 40 °C. Mobile phase A was water containing 0.1% formic acid, and mobile phase B was acetonitrile containing 0.1% formic acid. The flow rate was 0.30 mL/min and the injection volume was 7.5 μL. The gradient was programmed as follows: 0–1 min, 98% A/2% B; 1–9 min, a linear change to 2% A/98% B; 9–12 min, 2% A/98% B; 12-12.1 min, return to 98% A/2% B; and 12.1–15 min, re-equilibration at 98% A/2% B. Samples were analyzed in randomized order with intermittent quality control (QC) samples to monitor analytical stability.

Mass-spectrometric data were acquired using an LTQ-Orbitrap Elite high-resolution mass spectrometer (Thermo Fisher Scientific) equipped with an electrospray ionization source. Positive- and negative-ion data were acquired separately in Full MS-ddMS2 mode over m/z 70-1050. The MS1 resolution was 70,000, the MS2 resolution was 17,500, the automatic gain control target was 1 × 10^6, and the five most intense precursor ions were selected for data-dependent MS2 acquisition (TopN = 5). Stepped normalized collision energies of 30, 40, and 50 were used. In positive-ion mode, the ion-source heater temperature was 320 °C, sheath gas was 35 arbitrary units, auxiliary gas was 15 arbitrary units, sweep gas was 0 arbitrary units, spray voltage was 3.8 kV, capillary temperature was 350 °C, and the S-Lens radiofrequency level was 30%. In negative-ion mode, the corresponding settings were 320 °C, 35 arbitrary units, 15 arbitrary units, 1 arbitrary unit, 3.2 kV, 350 °C, and 60%, respectively.

Raw LC-MS data were processed using Compound Discoverer version 3.3 (Thermo Fisher Scientific) with the metabolomics workflow template. MS1 and MS2 spectra were matched against mzCloud and ChemSpider for compound annotation. The verified MS1 feature/compound-matching criterion was an absolute mass error of <5 ppm; the retention-time tolerance was <1 min and the intensity threshold was >1,000. Feature intensities exported from Compound Discoverer 3.3 were normalized by total ion current (TIC), log-transformed, and filtered using quality-control samples to remove features with poor reproducibility (QC relative standard deviation >30%). The normalized peak table was then Pareto-scaled for downstream multivariate analysis in SIMCA-P version 14.0. This single sequential workflow was used for metabolomics preprocessing and statistical analysis.

Unsupervised principal component analysis (PCA) was used to assess data structure and QC clustering. Supervised partial least-squares discriminant analysis (PLS-DA) and orthogonal partial least-squares discriminant analysis (OPLS-DA) models were used for exploratory multivariate visualization and group-discrimination assessment. OPLS-DA performance was summarized using model-fit and predictive parameters (R2X, R2Y, and Q2), and overfitting was evaluated separately by 200-iteration permutation testing. The permutation R2 and Q2 values were interpreted as validation statistics rather than as the fitted OPLS-DA model parameters. Candidate metabolic features were screened using variable importance in projection (VIP) > 1.0, nominal p < 0.05, and FC > 1.5 or FC < 0.67. Benjamini-Hochberg false discovery rate (FDR)-adjusted p values were calculated and are reported in **Table 1**; FDR was reported for transparency but was not used as a feature-selection criterion. Volcano plots and heatmaps were generated in R (ggplot2/pheatmap). Metabolite annotation was reported according to the Metabolomics Standards Initiative (MSI): Level 1 for compounds confirmed by authentic standards, Level 2 for MS/MS spectral matching, and Level 3 for putative annotations based on accurate mass or class-level evidence. Machine-learning analyses using Random Forest and support vector machine (SVM) models were treated as exploratory feature-ranking analyses given the small sample size.

**Table 1:** Putatively annotated serum features meeting the exploratory screening criteria in SCOD compared with controls.

Name	VIP	FC	log2 (FC)	p.value	FDR
Fludarabine	2.817	4.537	2.182	3.5529E-06	0.002537
Glycerophosphocholine	2.344	1.803	0.85	0.001673	0.23061
Adenosine 5’-diphosphoribose	2.328	6.724	2.749	0.00094	0.22662
N,n-dimethylsphingosine	2.306	1.518	0.602	0.001747	0.23061
1-palmitoyl-2-hydroxy-sn-glycero-3-phosphoethanolamine	2.304	1.525	0.609	0.00247	0.23061
Khasianine	2.287	2.01	1.008	0.002907	0.23061
Pro-Glu	2.133	0.481	-1.055	0.006079	0.36172
Zanamivir	2.118	1.653	0.725	0.007642	0.38353
Prostaglandin d1	2.103	0.12	-3.055	0.012217	0.46468
His-Glu	2.097	1.598	0.676	0.007374	0.38353
Wogonin	2.047	0.416	-1.266	0.004815	0.31256
Delsoline	2.006	0.497	-1.008	0.015093	0.47961
Leiocarposide	1.958	0.447	-1.161	0.010865	0.45633
Ribothymidine	1.928	3.891	1.96	0.021996	0.47961
Valine conjugated chenodeoxycholic acid	1.91	1.778	0.83	0.023756	0.47961
2-arachidonoylglycerol	1.905	2.106	1.074	0.014513	0.47961
(s)-perillic acid	1.893	0.527	-0.924	0.024136	0.47961
Arachidonoylserinol	1.889	1.65	0.723	0.014887	0.47961
Curcumol	1.883	0.489	-1.033	0.024854	0.47961
N-oleoylethanolamine	1.877	1.804	0.851	0.012365	0.46468
Tyr-Val	1.86	1.533	0.617	0.021776	0.47961
3’-azido-3’-deoxythymidine 5’-monophosphate	1.759	0.352	-1.506	0.026238	0.48793
Sulindac	1.759	2.805	1.488	0.030158	0.48793
Azelaic acid	1.751	1.813	0.858	0.041159	0.4886
Docosahexaenoic acid methyl ester	1.747	1.597	0.675	0.024687	0.47961
2-palmitoyl-rac-glycerol	1.73	1.999	0.999	0.030453	0.48793
20-Hydroxyarachidonic acid	1.729	0.395	-1.34	0.03955	0.4886
1-Stearoyl-2-oleoyl-sn-glycerol 3-phosphocholine (SOPC)	1.726	2.047	1.034	0.049975	0.4886
Dl-2,4-diaminobutyric acid	1.724	1.511	0.596	0.028588	0.48793
Isonerylgeraniol-18-oic acid	1.716	0.468	-1.097	0.047392	0.4886
Val-Val	1.714	1.575	0.655	0.040506	0.4886
Melezitose	1.704	1.928	0.947	0.042649	0.4886
Glycodeoxycholic acid	1.687	0.416	-1.266	0.023798	0.47961
5-aminovaleric acid betaine	1.683	1.532	0.615	0.040444	0.4886
Pepstatin a	1.683	1.965	0.974	0.044024	0.4886
Herbimycin a	1.675	0.377	-1.408	0.044374	0.4886
Gly-Pro-Ala	1.669	0.211	-2.247	0.047537	0.4886
Hexanoyl-l-carnitine	1.651	1.59	0.669	0.034593	0.4886
Glyceryl tripalmitoleate	1.645	3.645	1.866	0.042149	0.4886
1,5-pentanediamine	1.639	1.626	0.701	0.049316	0.4886
(+)-chlorpheniramine	1.636	0.382	-1.39	0.042708	0.4886
Acetyl-DL-Valine	1.58	1.828	0.87	0.047882	0.4886
Dl-malic acid	1.569	1.645	0.719	0.047144	0.4886

Features were selected using VIP > 1.0, nominal p < 0.05, and FC > 1.5 or FC < 0.67. FDR values are Benjamini-Hochberg-adjusted p values calculated across all 714 tested features. Compound identities are database-based putative annotations and require confirmation with authentic standards.

### Metagenomic sequencing procedures

Twenty-four hours before endpoint fecal collection, each mouse was transferred from its home cage to an individual clean metabolic cage. Fecal samples were collected separately from each mouse over the subsequent 24 h, preventing mixing or cross-contamination of fecal material among animals and ensuring that each sample could be attributed to an individual mouse. Following collection, the samples were placed in sterile cryotubes and immediately frozen at -80 °C. Fecal samples from 6 individual mice (3 SCOD model mice and 3 controls) were selected for metagenomic sequencing on the basis of sufficient fecal material and sample quality. DNA was extracted using the cetyltrimethylammonium bromide (CTAB) method.

Sequencing libraries were prepared using the NEBNext Ultra DNA Library Prep Kit for Illumina (NEB, USA; catalog no. E7370L) according to the manufacturer’s instructions, and index sequences were added to each sample. Genomic DNA was fragmented by sonication to approximately 350 bp, followed by end repair, A-tailing, adapter ligation, and PCR amplification. PCR products were purified using the AMPure XP system (Beverly, USA). Library quality was assessed using an Agilent 5400 system (Agilent, USA), and libraries were quantified by quantitative PCR. Qualified libraries were pooled at an effective concentration of 1.5 nM and sequenced on an Illumina platform using a paired-end 150-bp strategy.

Raw reads were evaluated with FastQC v0.12.1 and processed in a single quality-control workflow using KneadData (v0.12.2) with Trimmomatic (v0.39) for adapter removal (ILLUMINACLIP:adapters_path:2:30:10), sliding-window quality trimming (SLIDINGWINDOW:4:20), and removal of reads shorter than 50 bp (MINLEN:50). Host decontamination was performed by aligning reads to the mouse reference genome with Bowtie2 (v2.3.5.1) using the --very-sensitive preset; host-derived reads were removed before downstream analyses.

Primary taxonomic profiling was performed using Kraken2 (v2.0.7-beta) with a bacterial-focused local database containing approximately 16,799 known bacterial genomes and a confidence threshold of 0.2, followed by Bracken (v2.0) abundance estimation at different taxonomic levels using default parameters. This bacterial-focused workflow did not provide a comprehensive survey of fungal, archaeal, or viral communities.

Clean non-host reads were mapped to UniRef90 using HUMAnN3 (v3.6) with DIAMOND (v0.9.14) to obtain gene-family and pathway-abundance tables. The translated-search settings were a query coverage threshold of 90%, a prescreen threshold of 0.01, an E-value threshold of 1.0, and a subject coverage threshold of 50%. Species and functional abundance tables were used for clustering, principal-coordinate analysis (PCoA), non-metric multidimensional scaling (NMDS), linear discriminant analysis effect size (LEfSe) screening, and Dunn test comparisons where applicable. Antibiotic-resistance genes were annotated against the Comprehensive Antibiotic Resistance Database (CARD) using DIAMOND (v0.9.14) with E-value < 1e-3, percent identity > 80%, and --top 3.

Differential abundance analysis of microbial genes was performed using DESeq2 (v1.48.0) under Bioconductor (v3.21), with Benjamini-Hochberg false discovery rate (FDR) correction reported where available. Gene Ontology (GO) enrichment analysis of differentially abundant genes was conducted using clusterProfiler (v4.16.0) with GO.db (v3.21.0). Pathway annotation and enrichment were based on MetaCyc (v29.0) and Pathway Tools v29.0. Terms or pathways not supported after multiple-testing correction were treated as exploratory functional signals.

Statistical correlation analysis was performed to identify exploratory associations between altered gut microbial features and differential serum metabolites in SCOD versus control groups. Bacterial abundance and metabolite concentrations were log-transformed and normalized, and Spearman correlation coefficients were calculated for pairwise feature combinations. Nominal p values and adjusted p values were generated where available, and significant correlations were visualized as heatmaps in R. Because of the small metagenomic sample size and the large number of pairwise comparisons, these correlations were interpreted only as candidate associations requiring independent validation.

## Results

### Behavioral assessment

**Figure 1** suggests that repeated low-dose sevoflurane exposure was associated with impaired spatial learning and memory in SCOD model mice. During visible-platform testing (Day 1), swimming speeds were comparable between groups, which reduced the likelihood of gross motor impairment; however, swimming speed alone cannot exclude all motor, motivational, sensory, or stress-related influences on Morris water maze performance. During hidden-platform acquisition, SCOD mice showed longer escape latencies across training days, and during the probe trial they spent less time in the target quadrant.

**Figure 1:**
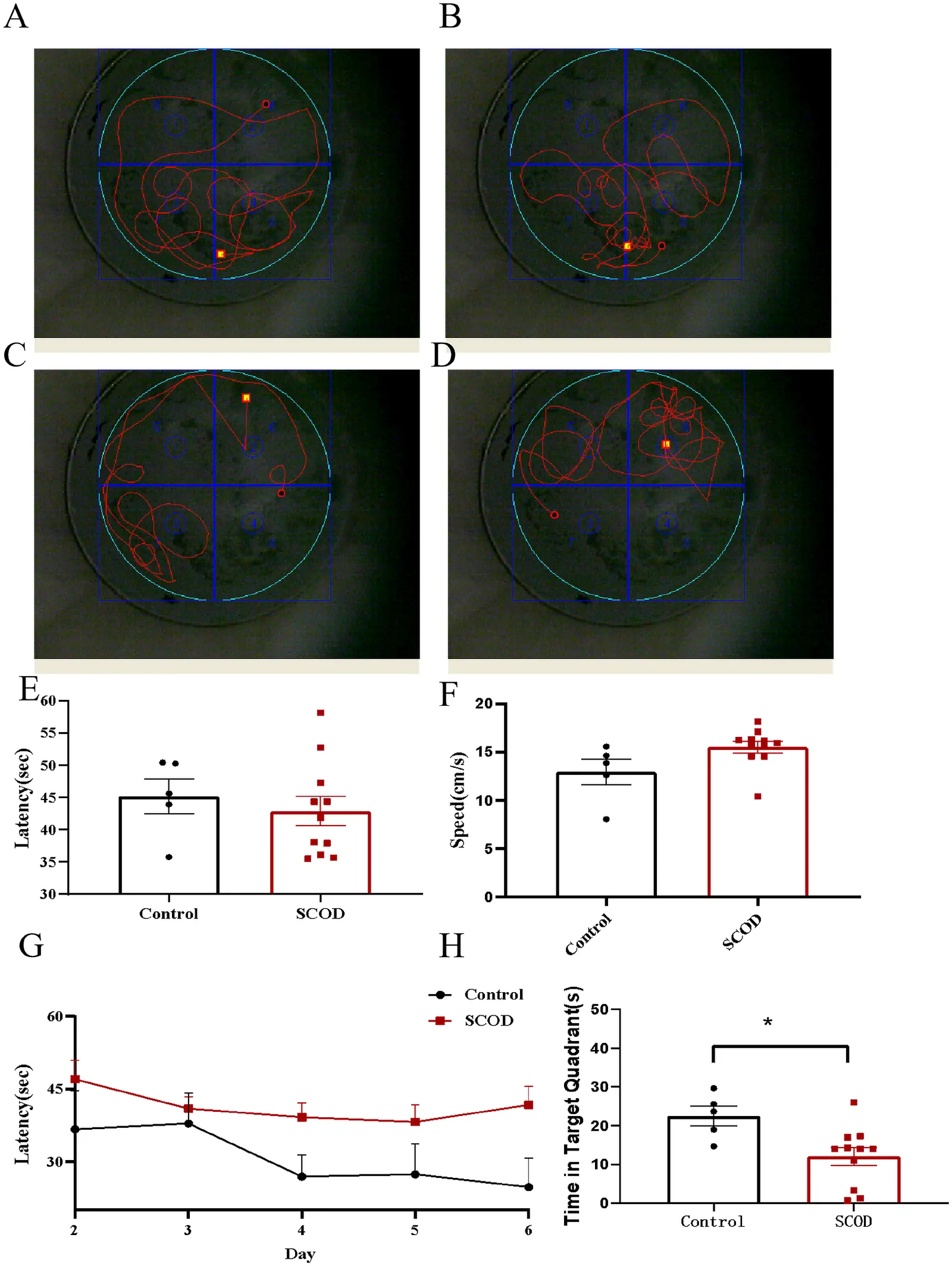
Effects of Sevoflurane on learning and memory function in mice. Behavioral performance in the Morris water maze task. **(A)** Representative swimming path of a normal mouse (control group). **(B)** Swimming path of an SCOD model mouse on day 1. **(C)** Representative swimming path of a normal mouse (control group). **(D)** Swimming path of an SCOD model mouse on day 7. **(E)** Latency to reach the platform in the visible platform test (day 1) for control and SCOD model groups. **(F)** Swimming speed in the visible platform test for control and SCOD model groups. **(G)** Latency to reach the platform in the hidden platform test for control and SCOD model groups. **(H)** Time spent in the target quadrant during the probe trial (platform removed) for control and SCOD model groups. * indicates a statistically significant difference between the control and SCOD groups (p < 0.05).

During visible-platform training (Day 1), both NS and SCOD groups showed similar escape latencies (NS: 25.3 ± 3.1 s; SCOD: 28.7 ± 2.8 s; p = 0.12) and swimming speeds (NS: 18.4 ± 1.2 cm/s; SCOD: 17.9 ± 1.5 cm/s; p = 0.45). In the hidden-platform phase (Days 2-6), daily means were calculated by averaging the four trials for each mouse within each day before repeated-measures analysis. The NS group showed progressive improvement in escape latency, whereas the SCOD group displayed prolonged latencies by Days 5-6 (Day 6: 28.6 ± 3.4 s vs. 12.5 ± 1.8 s in controls; p < 0.01). During the Day 7 probe trial, NS mice spent more time in the target quadrant (32.4 ± 2.9 s) than SCOD mice (18.7 ± 2.1 s; p < 0.001). The repeated-measures analysis incorporated mouse-level daily means across Days 2-6; the Day 6 contrast is reported as a representative late-acquisition comparison. Behavioral sample sizes were unequal (control n = 5; SCOD n = 10).

### Differential metabolite profiling in SCOD

Non-targeted serum metabolomics revealed group-associated metabolic differences between SCOD and controls. The OPLS-DA score plot showed apparent group separation (**Figure 2A**). The fitted model parameters were R2X = 0.595, R2Y = 0.992, and Q2 = 0.641, indicating apparent exploratory model fit and predictive performance within this dataset. In the 200-iteration permutation validation, the regression intercepts were R2 = 0.755 and Q2=-0.584 (**Figure 2B**); the negative Q2 intercept indicated that the permuted models did not outperform the original model and therefore did not support overfitting.

**Figure 2:**
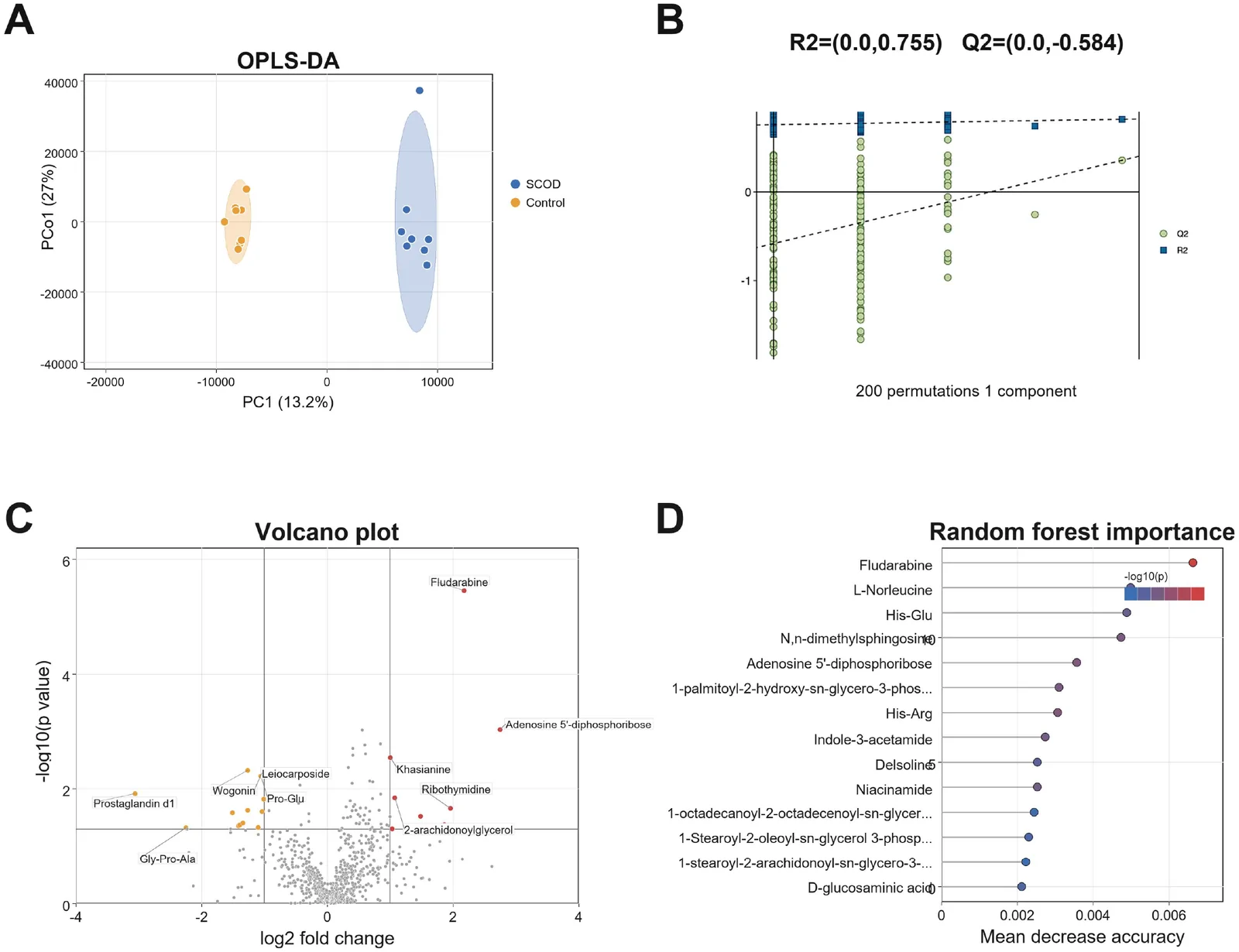
Multivariate analysis of metabolic profiles. **(A)** OPLS-DA score plot showing group separation (SCOD vs Control samples). Model-fit parameters were R2X = 0.595, R2Y = 0.992, and Q2 = 0.641. **(B)** Permutation assessment of the OPLS-DA model (200 iterations). Dashed lines indicate the regression intercepts for R2 (green) and Q2 (blue). The intercepts were R2 = 0.755 and Q2=-0.584 and are validation statistics rather than fitted-model parameters. **(C)** Volcano plot of metabolic features. The visualization threshold (|log2FC| > 1.0 and -log10(p) > 1.3) was used to emphasize large-effect nominal features. **Table 1** used VIP > 1.0, nominal p < 0.05, and FC > 1.5 or FC < 0.67; FDR values are reported in the table but were not used for feature selection. Red points denote features increased in SCOD. **(D)** Random Forest feature-importance ranking of candidate metabolites. The x-axis shows mean decrease in accuracy, and point color represents -log10(p).

Forty-three putatively annotated serum features met the exploratory screening criteria (VIP > 1.0, nominal p < 0.05, and FC > 1.5 or FC < 0.67), with 29 increased and 14 decreased in SCOD (**Table 1**). The corrected feature set included nominal annotations related to glycerophospholipids, sphingolipids, nucleotide metabolism, fatty-acid signaling, and bile acids, including glycerophosphocholine, adenosine 5’-diphosphoribose, N,N-dimethylsphingosine, prostaglandin D1, 2-arachidonoylglycerol, N-oleoylethanolamine, glycodeoxycholic acid, and hexanoyl-L-carnitine.

The volcano plot in **Figure 2C** uses a graphic threshold of |log2FC| > 1.0 to highlight large-effect features for visual clarity. This visualization cutoff is more stringent than the FC thresholds used for **Table 1** (FC > 1.5 or FC < 0.67, approximately log2FC > 0.585 or log2FC < -0.578). The volcano plot uses nominal p values for visualization, whereas **Table 1** additionally requires VIP > 1.0 and reports FDR for all 43 entries. Random Forest analysis provided an exploratory ranking of candidate metabolites in **Figure 2D**. Because no independent test set, external validation cohort, or formal feature-stability analysis was available, this ranking was not treated as a validated biomarker panel. **Table 2** reports the separate support vector machine feature-ranking results;

**Table 2:** Exploratory support vector machine (SVM) feature-ranking results.

Name	Rank Freq.	Importance	t.stat	p.value	FDR
Tyr-Ala	0.32	1.38E-04	3.23	8.70E-03	3.88E-01
Niacinamide	0.3	1.38E-04	-4.01	2.61E-03	2.31E-01
Tyr-Val	0.22	1.24E-04	-2.59	2.18E-02	4.80E-01
Indole-3-acetamide	0.12	1.14E-04	-3.80	2.01E-03	2.31E-01
Adenosine 5’-diphosphoribose	0.08	1.10E-04	-4.21	9.40E-04	2.27E-01
Pyridine	0.08	1.04E-04	-1.90	8.00E-02	5.26E-01
Gly-Lys	0.06	9.86E-05	-2.01	6.46E-02	5.09E-01
Pro-Glu	0.1	9.63E-05	3.23	6.08E-03	3.62E-01
D-glutamine	0.06	9.37E-05	-2.64	2.05E-02	4.80E-01
Gly-Pro-Ala	0.04	8.48E-05	2.24	4.75E-02	4.89E-01
Valine conjugated chenodeoxycholic acid	0.06	8.30E-05	-2.57	2.38E-02	4.80E-01
Arginine	0.08	8.03E-05	-1.70	1.12E-01	5.54E-01
2-docosahexaenoyl-1-palmitoyl-sn-glycero-3-phosphoethanolamine	0.06	7.50E-05	-1.82	9.11E-02	5.33E-01
1-palmitoyl-sn-glycero-3-phosphocholine	0.08	6.90E-05	-3.45	4.29E-03	3.06E-01
1-stearoyl-2-docosahexaenoyl-sn-glycero-3-phosphocholine	0.02	6.57E-05	-2.68	1.88E-02	4.80E-01
12s-hydroxy-5z,8e,10e-heptadecatrienoic acid	0.02	6.36E-05	1.70	1.12E-01	5.54E-01
Adenosine 5’-monophosphate	0.02	6.06E-05	1.67	1.19E-01	5.62E-01
Betaine	0.04	5.99E-05	-2.07	6.39E-02	5.09E-01
1-palmitoyl-2-hydroxy-sn-glycero-3-phosphoethanolamine	0.02	5.73E-05	-3.68	2.47E-03	2.31E-01
L-Norleucine	0.02	5.42E-05	-3.28	8.06E-03	3.84E-01
1-Stearoyl-2-oleoyl-sn-glycerol 3-phosphocholine (SOPC)	0.06	5.29E-05	-2.32	5.00E-02	4.89E-01
1-palmitoyl-2-linoleoyl-sn-glycero-3-phosphocholine	0.02	5.02E-05	-0.50	6.23E-01	8.60E-01
Gly-Gly	0.02	4.50E-05	1.18	2.67E-01	7.03E-01
9,12-octadecadiynoic acid	0.02	4.45E-05	1.58	1.38E-01	5.97E-01
Hexanoyl-l-carnitine	0.02	4.29E-05	-2.36	3.46E-02	4.89E-01
D-glucosaminic acid	0.02	3.91E-05	-2.55	3.17E-02	4.89E-01
Cytidine 5’-diphosphocholine	0.02	3.81E-05	0.66	5.24E-01	8.12E-01
Zearalenone	0.02	3.46E-05	1.06	3.06E-01	7.15E-01
N-(octadecanoyl)sphing-4-enine-1-phosphocholine	0.02	3.31E-05	-0.76	4.68E-01	8.01E-01
DL-phenylalanine	0.02	2.99E-05	-0.93	3.70E-01	7.38E-01
Glutathione	0.02	2.76E-05	-0.31	7.64E-01	9.21E-01
N-.alpha.-acetyl-l-ornithine	0.02	2.67E-05	0.34	7.40E-01	9.06E-01
Val-Trp	0.02	1.41E-05	0.11	9.13E-01	9.65E-01
Met-Met-Arg	0.02	1.00E-05	-1.02	3.26E-01	7.15E-01

None of the listed features met FDR < 0.05; the ranking is descriptive and should not be interpreted as a validated biomarker panel.

### Microbial community shifts

Metagenomic analysis revealed exploratory microbial compositional shifts between SCOD and control fecal samples across multiple taxonomic levels. Because only three individual mice per group were sequenced, these patterns should be considered preliminary and sensitive to individual and cage-related variation. At the phylum level, SCOD samples showed a lower relative *Bacteroidota*/*Bacillota* (historically *Bacteroidetes*/*Firmicutes*) ratio and lower *Verrucomicrobiota* abundance (**Figure 3A**). Because these data are compositional and based on relative abundances, this ratio should not be interpreted as direct evidence of absolute increases or decreases in either bacterial group. Class-, order-, and family-level comparisons suggested additional shifts in *Bacteroidia*, *Clostridia*, *Burkholderiales*, *Prevotellaceae*, and *Enterobacteriaceae* (**Figures 3B–D**).

**Figure 3:**
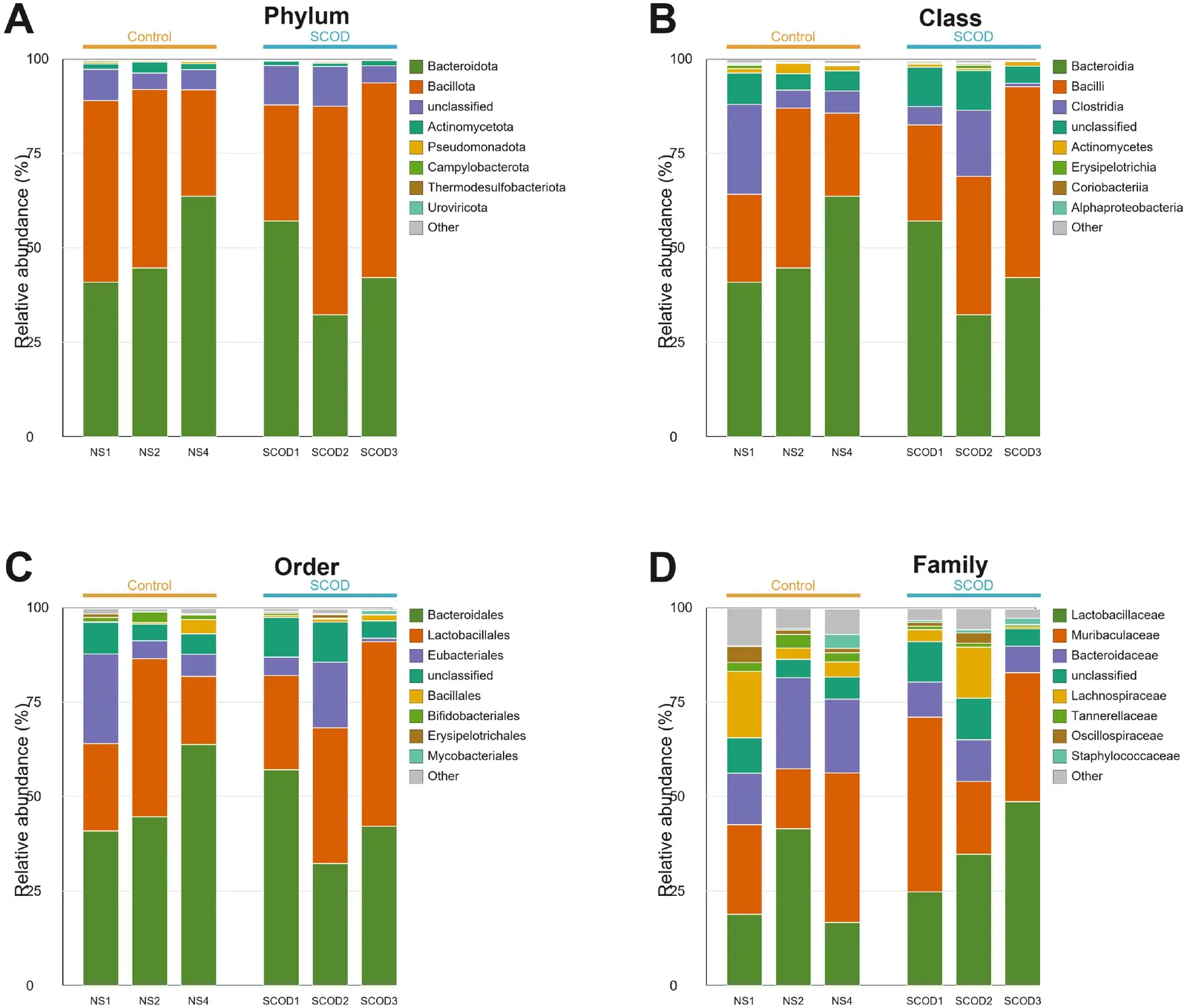
Comparative analysis of gut microbiota relative abundance in control and SCOD groups across taxonomic levels. **(A)** Phylum-level composition. **(B)** Class-level composition. **(C)** Order-level composition. **(D)** Family-level composition. Bars show relative abundance (%) for individual fecal samples, with samples grouped as control and SCOD.

Genus- and species-level relative-abundance profiles showed substantial between-animal variation (**Supplementary Figure 1**). Given the metagenomic sample size of three mice per group, these composition plots are presented descriptively and are not treated as independent evidence of differential abundance. LEfSe analysis (**Figure 4**) identified candidate differentially abundant taxa between SCOD and control samples across phylum, class, family, genus, and species levels (LDA > 2, p < 0.05). Given the limited metagenomic sample size, these taxa are described as exploratory candidates rather than robust microbial biomarkers.

**Figure 4:**
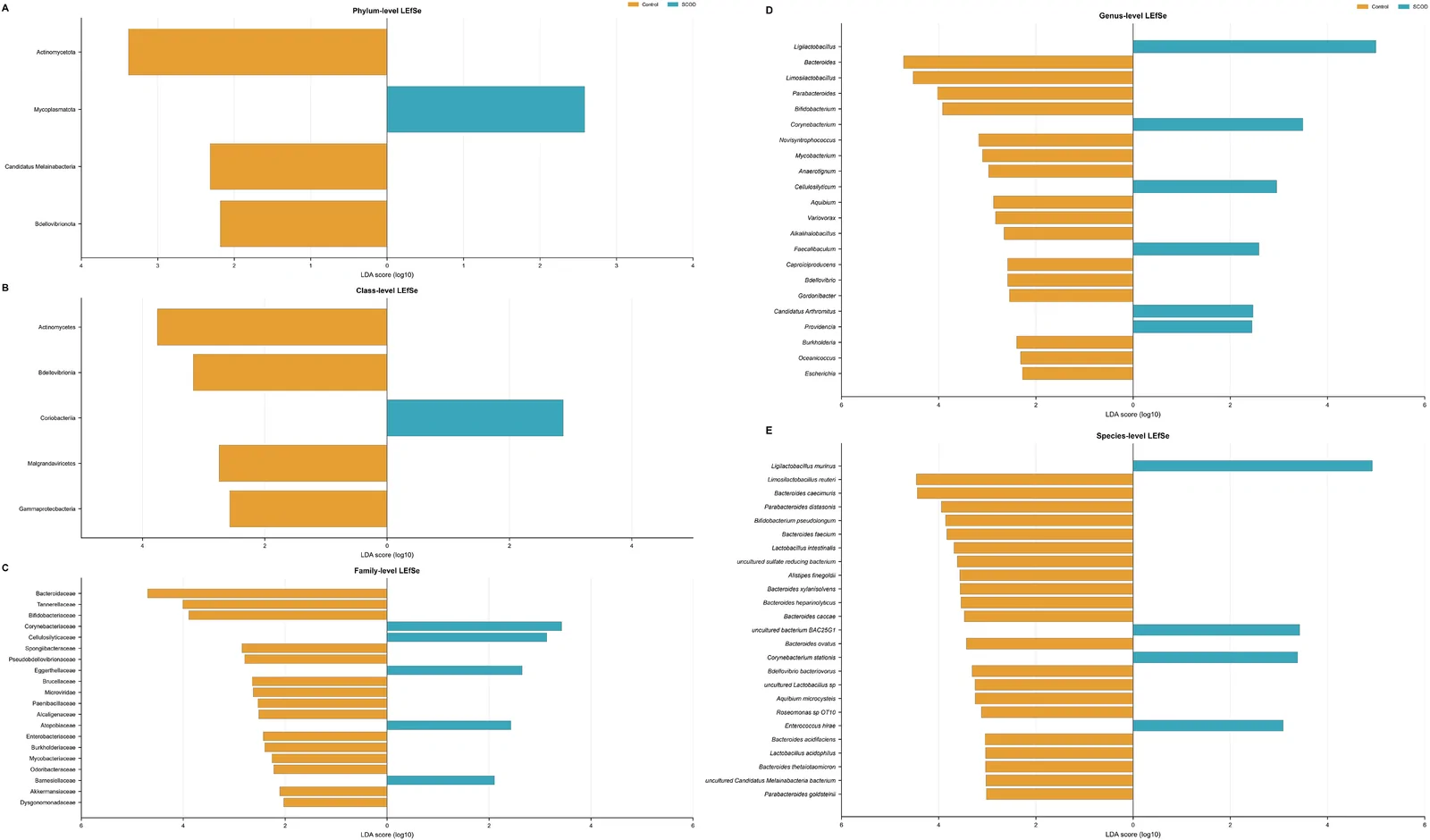
Candidate microbial taxa identified by exploratory LEfSe analysis. **(A)** Phylum-level results. **(B)** Class-level results. **(C)** Family-level results. **(D)** Genus-level results. **(E)** Species-level results. Orange bars indicate candidates enriched in controls and teal bars indicate candidates enriched in SCOD (LDA > 2, nominal p < 0.05). Only taxa assigned to the stated rank are displayed. With n = 3/group, these results are hypothesis-generating rather than validated biomarkers.

At the species level, LEfSe identified *Ligilactobacillus murinus*, *Corynebacterium stationis*, and *Enterococcus hirae* among the SCOD-enriched candidates, whereas *Limosilactobacillus reuteri*, *Bacteroides caecimuris*, and *Parabacteroides distasonis* were among the control-enriched candidates (**Figure 4E**). These nominal signals require validation in larger, cage-aware cohorts and independent taxonomic confirmation. GO enrichment of differential microbial features highlighted candidate functional terms related to signal transduction, hydrolase/peptidase activity, transport, histidine catabolism, dinitrosyl-iron complex binding, nicotinamide nucleotide metabolism, and ADP-L-glycero-beta-D-manno-heptose biosynthesis (**Figure 5**). The dot plot reports GeneRatio, Count, and adjusted p value (p.adjust) for displayed terms.

**Figure 5:**
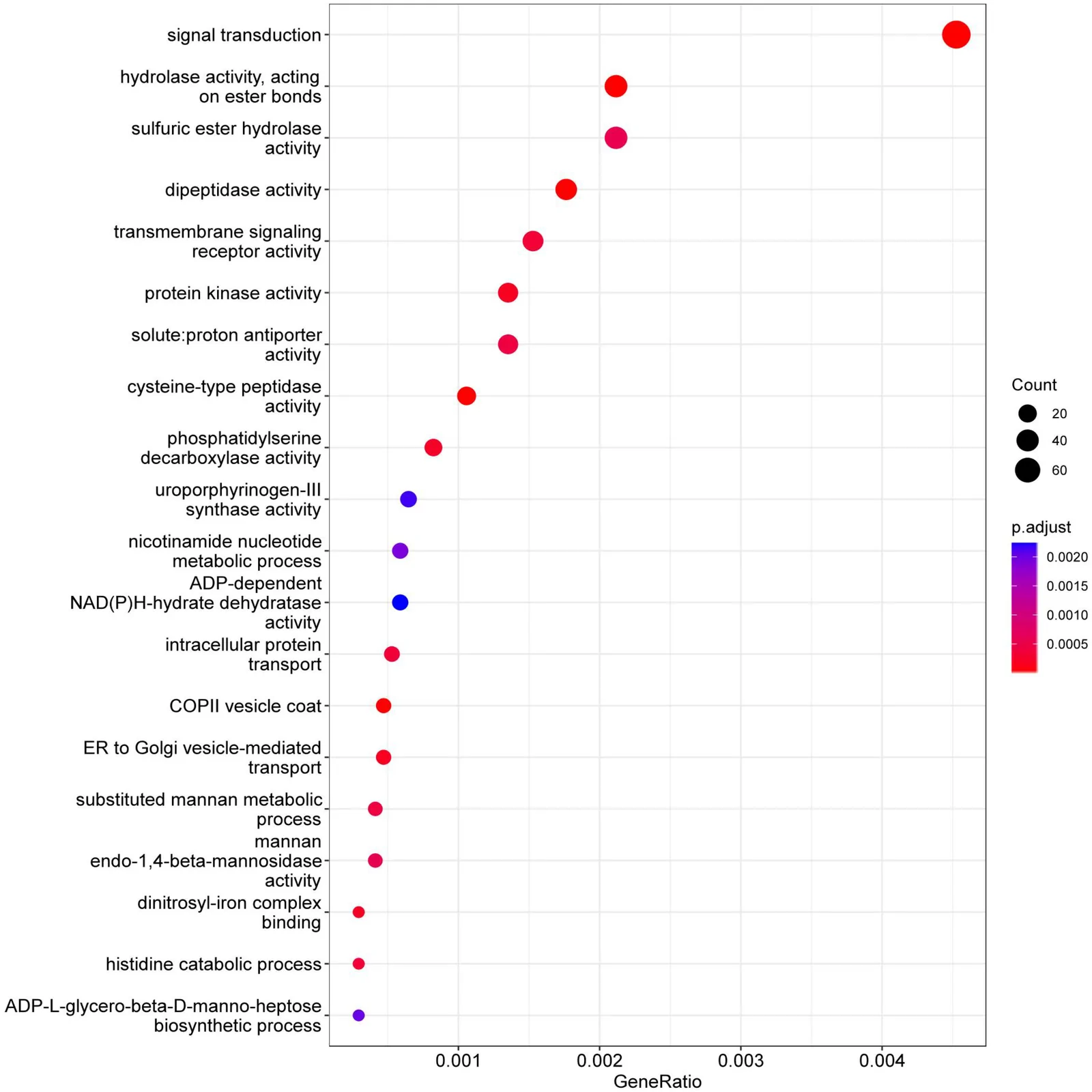
GO enrichment dot plot of candidate microbial functional terms. The x-axis shows GeneRatio, dot size represents Count, and color represents adjusted p value (p.adjust). Displayed terms include signal transduction, hydrolase activity, sulfuric ester hydrolase activity, dipeptidase activity, transmembrane signaling receptor activity, histidine catabolic process, dinitrosyl-iron complex binding, nicotinamide nucleotide metabolic process, and ADP-L-glycero-beta-D-manno-heptose biosynthetic process.

Functional composition summaries further compared selected GO categories and MetaCyc pathway categories between SCOD and control samples (**Figure 6**). At the GO level, the displayed categories accounted for relatively small proportions of annotated sequences compared with the broad ‘Other’ category (**Figure 6A**). At the pathway level, selected categories such as GLYCOGENSYNTH-PWY, peptidoglycan biosynthesis, amino-acid biosynthesis, and related biosynthetic pathways contributed small proportions of annotated sequences (**Figure 6B**).

**Figure 6:**
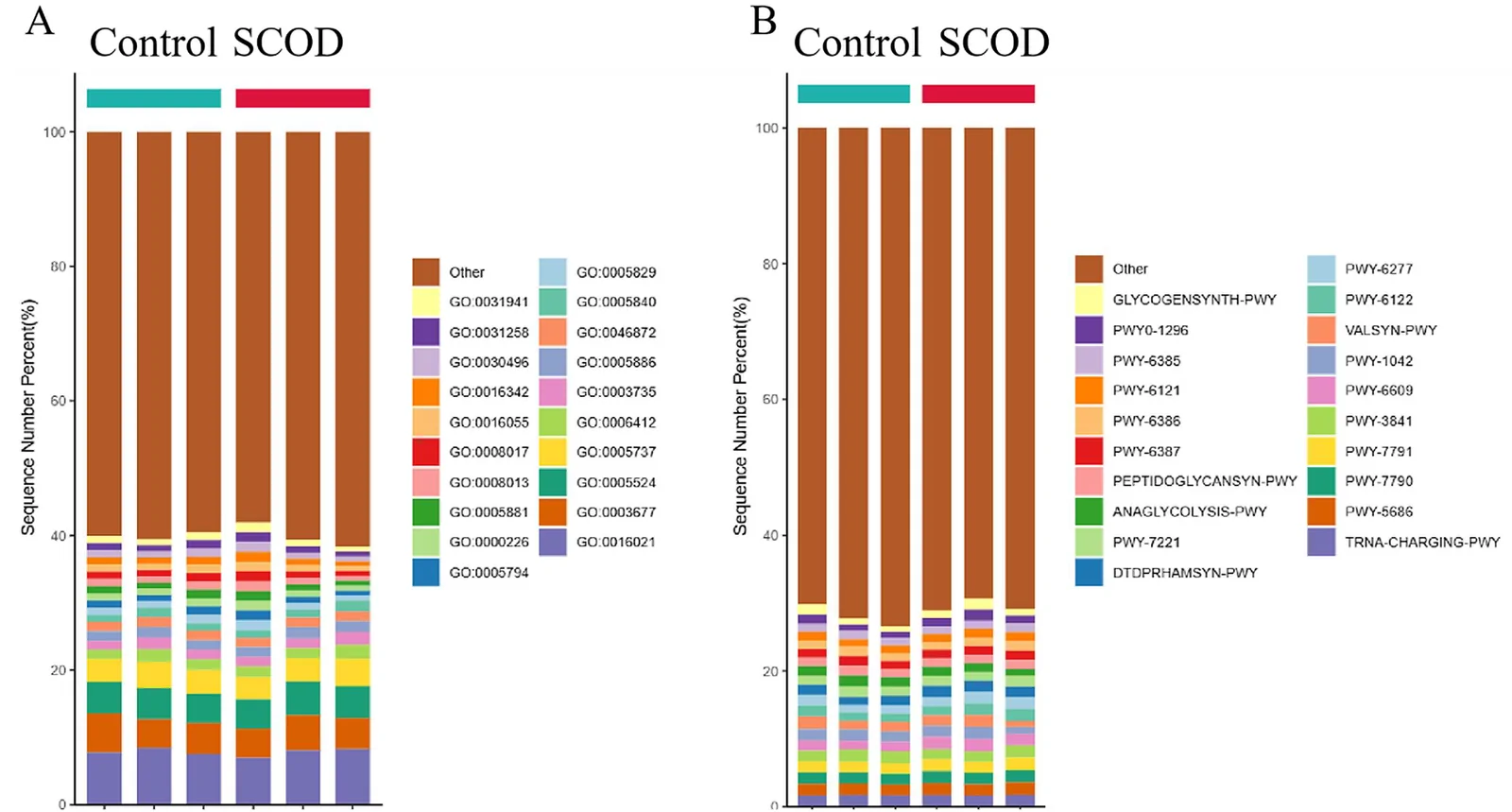
Functional composition profiles of selected microbial GO and pathway categories in control and SCOD samples. **(A)** Relative sequence-number percentages for selected GO categories. **(B)** Relative sequence-number percentages for selected MetaCyc pathway categories. Percent values indicate the relative contribution of each displayed category within annotated sequences rather than enrichment significance.

Integrated microbiota-metabolite networks revealed exploratory Spearman correlation patterns between microbial features and serum metabolites (**Figure 7**). Pairwise correlations were screened using nominal and adjusted p values where available, but the small metagenomic sample size makes these estimates unstable and sensitive to individual observations. These associations were used to generate hypotheses regarding microbiota-metabolite coupling but were not interpreted as evidence that specific taxa produced or regulated specific metabolites.

**Figure 7:**
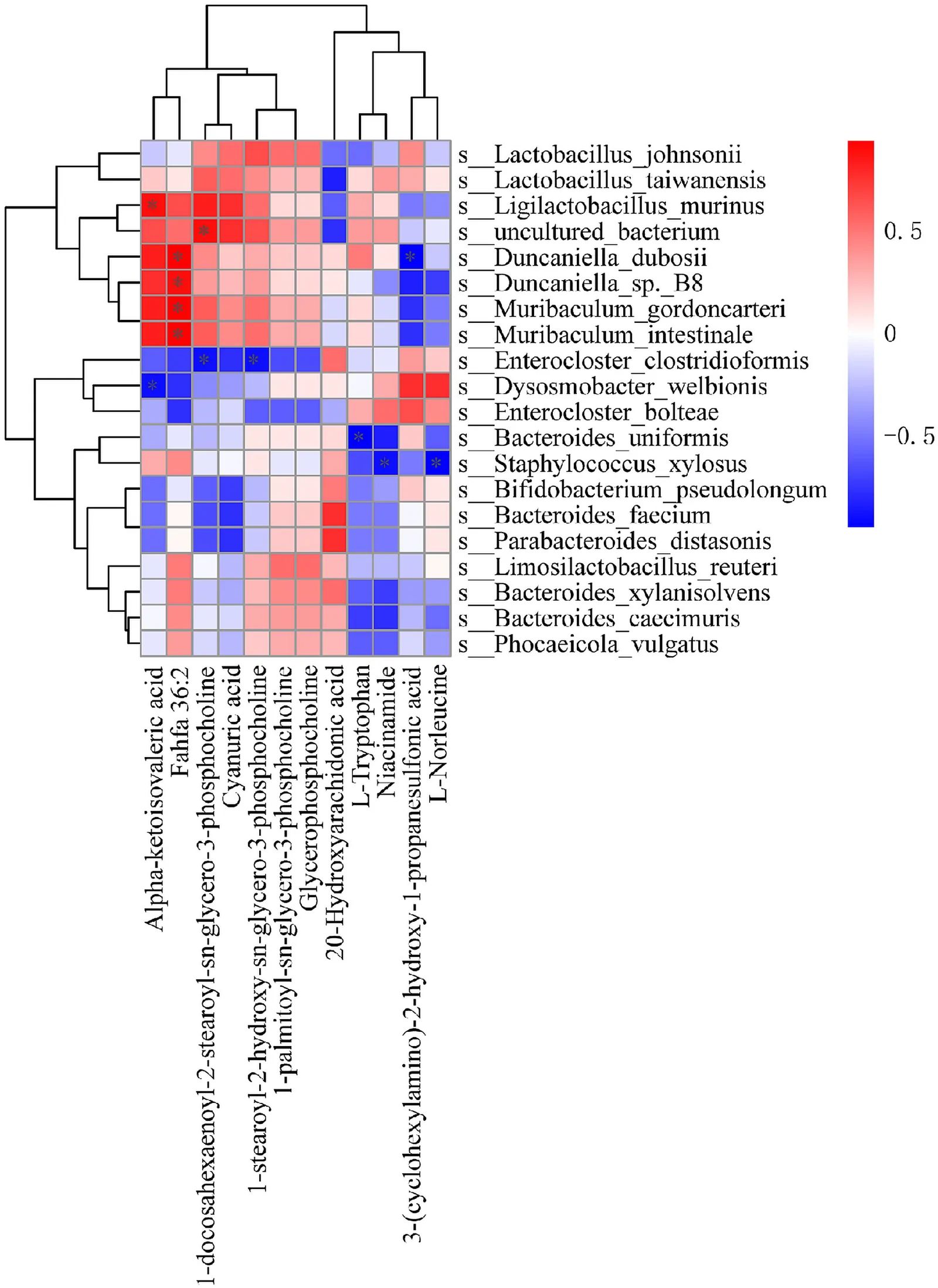
Correlation heatmap of differential metabolites and bacterial species between SCOD and control groups.

## Discussion

Inhalation anesthetics are indispensable for modern surgery, but low-level waste-gas exposure remains a concern in operating-room environments (Braz et al., 2017; Jafari et al., 2018; Emara et al., 2020; Ji et al., 2021; Oliveira et al., 2021). In this study, we used a repeated low-dose sevoflurane exposure model without surgery or injection stress to examine cognitive performance and multi-omics associations. The 7-day 100 ppm model was an upper-range experimental proof-of-concept exposure and does not reproduce routine well-scavenged operating-room concentrations, the full duration of occupational exposure, or the complexity of human workplace conditions. Therefore, the findings should be interpreted as experimental evidence of association and as a basis for future mechanistic testing rather than direct evidence of routine occupational health risk.

The behavioral results suggest spatial learning and memory impairment in the SCOD group, as reflected by delayed escape latencies during hidden-platform trials and reduced target-quadrant exploration during the probe test. Comparable swimming speeds on Day 1 reduced the likelihood that gross motor dysfunction explained the behavioral phenotype, but did not exclude all motivational, sensory, stress-related, or physiological influences. The closest repeated non-surgical studies remain substantially different from the present model: Ozer et al. used 0.3% sevoflurane for 4 h/day over 30 days (Ozer et al., 2006), Guo et al. used 1.5% or 2.5% for 2 h/day over 5 days in aged rats (Guo et al., 2018), and Liang et al. used 3% across repeated neonatal exposures (Liang et al., 2023). The latter two studies reported hippocampal BDNF/NF-kappaB/apoptosis or tau-phosphorylation changes, but those mechanisms cannot be assumed at 0.01% because they were not measured here. The unequal behavioral sample sizes, repeated chamber handling, and absence of additional neurophysiological endpoints further require cautious interpretation.

The reconstructed serum feature table contained putative annotations spanning glycerophospholipid, sphingolipid, nucleotide, fatty-acid signaling, and bile acid-related classes. These class-level patterns are therefore exploratory and do not establish pathway dysregulation, hippocampal metabolic changes, or central nervous system metabolic remodeling. Nominal features included N,N-dimethylsphingosine and several phosphocholine species. Broad literature on sphingolipid homeostasis in brain development and neurological disease provides biological context (Olsen and Faergeman, 2017), but these putative serum annotations did not meet FDR < 0.05. Blood-brain barrier (BBB) permeability, microglial activation, inflammatory cytokines, oxidative stress markers, and neuronal apoptosis were not measured. The lipid-related signals are therefore hypotheses for targeted validation rather than evidence of a sphingolipid-mediated mechanism.

Two bile acid-related putative annotations, valine-conjugated chenodeoxycholic acid and glycodeoxycholic acid. Human data associating altered bile acid profiles with cognitive impairment provide disease-level context (MahmoudianDehkordi et al., 2019), but do not establish a bile acid mechanism in repeated low-dose sevoflurane exposure. Targeted bile acid profiling, supplementation, and rescue experiments were not performed; no causal interpretation was assigned to these features. Machine-learning feature rankings identified candidate metabolic signals, including lipid and amino acid features, that may guide future validation. These findings should be interpreted as hypothesis-generating because the models were trained on a small dataset without an independent validation cohort or formal stability analysis; future targeted validation and external-cohort testing are required before clinical biomarker claims can be made.

Sevoflurane-specific microbiome studies provide model-dependent context for the taxonomic findings. A single anesthetic-range exposure produced time-dependent changes involving *Bacteroides*, *Alloprevotella*, *Akkermansia*, and *Lactobacillus* (Han et al., 2021). Repeated 3% exposure in aged mice was associated with reduced *Akkermansia* abundance and cognitive and inflammatory changes that were partly attenuated by probiotics (Han et al., 2024), whereas a repeated developmental model reported changes involving *Ligilactobacillus*, *Faecousia*, *Alloprevotella*, and *Lactobacillus* and attenuation with antibiotic pretreatment (Zhang et al., 2026). Differences in age, dose, sampling time, and intervention likely contribute to the differing taxonomic directions. These studies do not validate the species-level signals in the present n = 3/group dataset or show that any identified taxon mediates cognitive impairment at 100 ppm.

At a broad descriptive level, the present observation of microbial community shifts is consistent with direct sevoflurane-microbiome studies (Han et al., 2021; Han et al., 2024; Zhang et al., 2026). However, those studies used single or repeated anesthetic-range exposure in adult, aged, or developing animals, whereas the present study used repeated 0.01% exposure and did not measure NLRP3 activation, inflammatory cytokines, or brain pathology. The relative Bacteroidota/Bacillota (historically Bacteroidetes/Firmicutes) ratio and the candidate differences involving *Akkermansia* and other taxa therefore remain exploratory associations.

Several important limitations should be emphasized. First, causal experiments such as fecal microbiota transplantation, antibiotic-mediated microbiota depletion, targeted bacterial supplementation, bile acid rescue, sphingolipid pathway intervention, or metabolite rescue were not performed. Second, blood and brain sevoflurane levels and session-level chamber concentration summaries were not measured or retained, so internal exposure dose and exposure variability could not be confirmed formally. Third, FiO2 was maintained near 0.40 in both groups, but no separate ambient-air comparison group was included; the independent physiological contribution of supplemental oxygen therefore cannot be determined. Fourth, longitudinal body-weight values were not retained for formal group comparison. Fifth, brain tissue, cerebrospinal fluid, hippocampal molecular markers, BBB permeability, microglial activation, inflammatory cytokines, oxidative stress, and neuronal apoptosis were not assessed. Finally, fecal samples were collected separately after 24 h of individual housing in clean metabolic cages, which prevented fecal mixing and cross-contamination but did not eliminate microbiome similarities arising from the preceding shared home-cage environment. The small metagenomic sample size, two-home-cage-per-group design, unrecorded home-cage distribution of the sequenced animals, and lack of cage-effect modeling limit species-level, functional, and network-level conclusions. The microbiome differences and microbiota-metabolite correlations should therefore be interpreted as exploratory, hypothesis-generating findings.

Taken together, the microbiota-metabolite associations observed in this study provide a plausible framework for future studies of the gut-brain axis in repeated low-dose sevoflurane exposure. These associations do not prove microbial production of specific metabolites, metabolite-mediated neuronal injury, or causality between microbiota changes and cognitive impairment.

Future studies should incorporate larger cage-aware cohorts, an ambient-air comparison group, internal exposure measurements, targeted brain and hippocampal assays, and interventional designs such as fecal microbiota transplantation (FMT), microbiota depletion, bacterial supplementation, or metabolite rescue to test whether the candidate pathways identified here are necessary or sufficient for cognitive dysfunction. Targeted metabolomics and taxonomic assays are also needed to validate the candidate microbial and metabolic signals identified by untargeted approaches. Overall, repeated low-dose sevoflurane exposure in this proof-of-concept model was associated with cognitive impairment and exploratory microbiota-metabolite alterations, but the current design supports association rather than causality.

## Conclusions

This study suggests that repeated low-dose sevoflurane exposure, independent of surgery, is associated with impaired spatial learning and memory in mice. Because the 100 ppm, 2 h/day, 7-day exposure paradigm exceeds typical well-scavenged operating-room concentrations, the findings should be interpreted as deriving from an experimental proof-of-concept model rather than routine occupational exposure. Integrated serum metabolomics and shotgun metagenomics identified exploratory microbiota-metabolite associations, including bile acid-, sphingolipid-, and gut-microbial candidate signals. However, the current data are associative and hypothesis-generating. Direct causal roles for the gut microbiota, individual taxa, or specific metabolites require larger cage-aware studies, an ambient-air comparison group, targeted validation, and interventional experiments.

## Data Availability

All study data generated or analyzed during this study are available from the corresponding author upon reasonable request. The untargeted LC-MS metabolomics raw files and processed metabolomics data have been deposited in MetaboLights under accession number MTBLS15846. The metagenomic sequencing raw data have been deposited in NCBI under BioProject accession number PRJNA1505144.

## Supplementary material

Supplementary Figure 1 Descriptive gut microbiota composition in control and SCOD samples. **(A)** Genus-level relative-abundance profiles. **(B)** Species-level relative-abundance profiles. Bars represent individual fecal samples; no inferential comparison is shown in these panels.
