# Supplementary material for: Exploratory microbiota-metabolite alterations associated with cognitive impairment after repeated low-dose sevoflurane exposure in mice
Source: Front Cell Infect Microbiol. 2026 Sep 30;16:1869293. doi: 10.3389/fcimb.2026.1869293 (PMC13662922; doi:10.3389/fcimb.2026.1869293)
Supplement: Supplementary file 1 [file SupplementaryFile1.docx]

Exploratory Microbiota-Metabolite Alterations Associated with Cognitive Impairment after Repeated Low-Dose Sevoflurane Exposure in Mice

Hui Tang ^1，#^, Qingqing Xiong ^2，#^, Gang Qian ^1,*^

1.Department of Anesthesiology, Tongren Hospital, Shanghai Jiao Tong University School of Medicine, 1111 XianXia Road, Shanghai 200336, China

2.Department of Anesthesiology, Lijiang People's Hospital, No. 526, Fuhui Road, Gucheng District, Lijiang City, Yunnan Province, 674100, China

\# Both authors contributed equally to this work.

* Corresponding authors: Gang Qian，Department of Anesthesiology, Tongren Hospital, Shanghai Jiao Tong University School of Medicine, 1111 XianXia Road, Shanghai 200336, China；E-mail:drqiantr@sjtu.edu.cn.

Figure S1. Descriptive gut microbiota composition in control and SCOD samples.
(A) Genus-level relative-abundance profiles.
(B) Species-level relative-abundance profiles. Bars represent individual fecal samples; no inferential comparison is shown in these panels.
